# Virulence Patterns in a Murine Sepsis Model of ST131 *Escherichia coli* Clinical Isolates Belonging to Serotypes O25b:H4 and O16:H5 Are Associated to Specific Virotypes

**DOI:** 10.1371/journal.pone.0087025

**Published:** 2014-01-30

**Authors:** Azucena Mora, Ghizlane Dahbi, Cecilia López, Rosalía Mamani, Juan Marzoa, Sara Dion, Bertrand Picard, Miguel Blanco, María Pilar Alonso, Erick Denamur, Jorge Blanco

**Affiliations:** 1 Laboratorio de Referencia de *E. coli* (LREC), Departamento de Microbioloxía e Parasitoloxía, Facultade de Veterinaria, Universidade de Santiago de Compostela (USC), Lugo, Spain; 2 IAME, UMR 1137, INSERM, Paris, France; Univ Paris Diderot, Sorbonne Paris Cité, Paris, France; 3 IAME, UMR 1137, INSERM, Paris, France; Univ Paris Nord, Sorbonne Paris Cité, Paris, France; 4 Unidade de Microbioloxía Clínica, Hospital Universitario Lucus Augusti (HULA), Lugo, Spain; University of Maryland School of Medicine, United States of America

## Abstract

*Escherichia coli* sequence type (ST)131 is an emerging disseminated public health threat implicated in multidrug-resistant extraintestinal infections worldwide. Although the majority of ST131 isolates belong to O25b:H4 serotype, new variants with different serotypes, STs using the discriminative multilocus sequence typing scheme of Pasteur Institute, and virulence-gene profiles (virotypes) have been reported with unknown implications on the pattern of spread, persistence and virulence. The aim of the present study was to compare virulence in a mouse subcutaneous sepsis model of representative ST131 clinical isolates belonging to 2 serotypes (O25b:H4, O16:H5) and nine virotypes and subtypes (A, B, C, D1, D2, D3, D4, D5 and E). Fourteen out of the 23 ST131 isolates tested (61%) killed 90 to 100% of mice challenged, and 18 of 23 (78%) at least 50%. Interestingly, different virulence patterns in association with virotypes were observed, from highly rapid lethality (death in less than 24 h) to low final lethality (death at 7 days) but with presence of an acute inflammation. This is the first study to assess virulence of ST131 isolates belonging to serotype O16:H5, which exhibited virotype C. In spite of their low virulence-gene score, O16:H5 isolates did not show significant differences in final lethality compared with highly virulent O25b:H4 isolates of virotypes A, B and C, but killed mice less rapidly. Significant differences were found, however, between virotypes A, B, C (final lethality ≥80% of mice challenged) and virotypes D, E. Particularly unexpected was the low lethality of the newly assigned virotype E taking into account that it exhibited high virulence-gene score, and the same clonotype H30 as highly virulent O25b:H4 isolates of virotypes A, B and C. *In vivo* virulence diversity reported in this study would reflect the genetic variability within ST131 clonal group evidenced by molecular typing.

## Introduction

*Escherichia coli* O25:H4 sequence type (ST)131, associated with the CTX-M-15 extended-spectrum β-lactamase (ESBL), has emerged as a multidrug-resistant pathogen reported internationally [1]–[3].

Different authors have already described the heterogeneity within the clonal group ST131, not only on the basis of the virulence-gene content, the variety of ESBL enzymes produced, antibiotic resistance and pulsed-field gel electrophoresis (PFGE) profiles, but also for the number of reservoirs from which it has been isolated [4]–[9]. In fact, four main virotypes (A to D) have been recently described within isolates O25b:H4-B2-ST131 which showed to be internationally distributed, corresponded with specific PFGE clusters, and exhibited distinctive clinical-epidemiological associations [10]. This variability within ST131 has been also demonstrated using the discriminative multilocus sequence typing (MLST) scheme of Pasteur Institute [7], [8].

*In vivo* studies have reported that the great majority of *E. coli* isolates belonging to the genetic group B2 are highly virulent in a sepsis mouse model [11], [12]. Specifically, Clermont and colleagues suggested that the ST131 clone is highly virulent since, like other B2 isolates, it killed 100% of the mice challenged in this model [13]. Other studies have pointed away from ST131 as having higher virulence potential compared with other extraintestinal pathogenic *E. coli* [14], [15].

To get more insight into the virulence potential of clonal group ST131, and considering its heterogeneity, we used a mouse subcutaneous sepsis model [16] to assess the virulence of 23 ST131 *E. coli* clinical isolates belonging to O25b:H4 and O16:H5 serotypes, and representative of nine ST131 virotypes and subtypes (A, B, C, D1, D2, D3, D4, D5 and E).

## Materials and Methods

### Ethics Statement

All animal experimentation was conducted following European (Directive 2010/63/EU on the protection of animals used for scientific purposes) and National (RD 53/2013) regulations for transport, housing and care of laboratory animals. The protocol used was approved by the Animal Welfare Committe of the Veterinary Faculty in Lugo, University of Santiago de Compostela (AE-LU-002/12/INV MED.02/OUTROS 04).

Female RjOrl:Swiss mice (3–4 weeks old, 14–18 g) purchased from Janvier Labs (Saint Berthevin, France) were housed under standard conditions with water and food supplied *ad libitum*. All efforts were made to minimize suffering. After inoculation, mice were monitored and clinically inspected several times day and night within 1 week. Surviving mice were euthanatized on day 7 by cervical dislocation.

### *E. coli* Isolates

The 25 *E. coli* isolates used in this study included 23 ST131 Spanish human extraintestinal clinical isolates, mainly recovered from urinary tract infections (UTIs) or bacteremia, plus the commensal derived strain K-12 MG1655 (O16-A-ST98) and the urosepsis strain CFT073 (O6-B2-ST73) as negative and positive controls, respectively, for the murine infection [11].

The selection of the 23 representative ST131 isolates was performed based on the virotypes, which had been previously described [4], [8], [10] from a collection of 656 ST131 human clinical isolates (Table 1). Twenty-one of those 23 ST131 isolates had already been characterized with regard to antibiotic susceptibility and molecular resistance mechanisms, O:H serotypes, a reduced extraintestinal virulence-gene scheme, phylogenetic groups, STs according to the Achtman scheme using seven housekeeping genes (*adk, fumC, gyrB, icd, mdh, purA* and *recA*) (http://mlst.ucc.ie/mlst/dbs/Ecoli), and *Xba*I-PFGE profiles as referenced previously [4], [8], [10], [17], [18]. Characterization of the remaining 2 isolates (H2623 and FV 13998), as well as complete typing of the virulence-gene scheme for detection of 40 ExPEC-associated virulence genes by PCR, was performed as described elsewhere [10]. The genes we sought to detect included *fimH, fimAv_MT78_,* ISL*3*-like in *fimB*, F10 *papA*, *papAH*, *papC*, *papEF* (positive isolates are tested for *papG* I, *papG* II, *papG* III alleles), *sfa/focDE* (only positive isolates are tested for *sfaS* and *focG*), *afa/draBC, afa* operon FM955459, *iha, bmaE, gafD, sat, cdtB, cnf1, hlyA, iucD, iutA, iroN, fyuA, chuA, kpsM II* (establishing *neuC-*K1, K2 and K5 variants), *kpsM III, cvaC*, *iss*, *traT*, *ibeA*, *malX* (PAI), *usp*, *tsh,* and *ompT*. Isolates were classified as ExPEC if they carried ≥2 of *papEF* (P fimbriae), *sfa/focDE* (S/F1C fimbriae), *afa/draBC* (Afa/Dr adhesins), *iutA* (aerobactin receptor), and *kpsM II* (group 2 capsule synthesis) [19].

**Table 1:** Characteristics of the 23 ST131 extraintestinal *E. coli* clinical isolates studied.

Isolate code	Year	Province (Spain)	Clinic origin	Serotype	Phylogroup	STAtchman	Virotype	BLEEtype	Antibioticresistances^a^	Reference
FV 9873	2007	Lugo	UTI	O25b:H4	B2	131	A	CTX-M-15	NAL CIP SXT TOB	[17]
FV 7563	2006	Lugo	UTI	O25b:H4	B2	131	A	CTX-M-15	NAL CIP SXT TOB	[17]
35 BA	2008	Barcelona	UTI	O25b:H4	B2	131	B	CTX-M-15	NAL CIP SXT GEN TOB	[18]
25.27	2006	Madrid	Pneumonia (sputum)	O25b:H4	B2	131	B	CTX-M-15	NAL CIP SXT GEN TOB	[10]
20.22	2006	A Coruña	UTI	O25b:H4	B2	131	C	CTX-M-14, CTX-M-15	NAL CIP SXT GEN TOB AMC FOF	[10]
45.09	2006	Valencia	UTI	O25b:H4	B2	131	C	CTX-M-15	NAL CIP SXT GEN TOB AMC	[10]
28.65	2006	Madrid	UTI	O25b:H4	B2	131	D	CTX-M-14	NAL CIP SXT TOB	[10]
H1447	2004	Lugo	Bacteremia	O25b:H4	B2	131	D		NAL SXT	[4]
FV 11171	2008	Lugo	Bacteremia	O25b:H4	B2	131	D	CTX-M-9	SXT GEN TOB AMC	[4]
H2525	2008	Lugo	Bacteremia	O25b:H4	B2	131	D			[4]
H1659	2005	Lugo	Bacteremia	O25b:H4	B2	131	D			[4]
H2341	2008	Lugo	Bacteremia	O25b:H4	B2	131	D		SXT	[4]
FV 14025	2009	Lugo	UTI	O25b:H4	B2	131	D	CTX-M-9	NAL SXT	[4]
83 BA	2008	Barcelona	UTI	O25b:H4	B2	131	D	SHV-12	NAL	[18]
FV 17616	2012	Lugo	UTI	O25b:H4	B2	131	D	CTX-M-1	NAL CIP SXT AMC	[8]
61 BA	2008	Barcelona	Abscess (pus)	O25b:H4	B2	131	D	SHV-12	NAL CIP SXT	[18]
50 BA	2008	Barcelona	UTI	O25b:H4	B2	131	D	CTX-M-14	NAL SXT	[18]
FV17578	2012	Lugo	UTI	O25b:H4	B2	131	New	CTX-M-15	NAL CIP TOB	[8]
FV 17543	2012	Lugo	UTI	O25b:H4	B2	131	New	CTX-M-15	NAL CIP SXT	[8]
FV 17539	2012	Lugo	UTI	O16:H5	B2	131	C	CTX-M-14	NAL CIP SXT GEN TOB	[8]
FV 17598	2012	Lugo	UTI	O16:H5	B2	131	C	CTX-M-14	NAL CIP SXT GEN TOB	[8]
H2623	2009	Lugo	Bacteremia	O16:H5	B2	131	C		NAL CIP	This study
FV 13998	2009	Lugo	UTI	O16:H5	B2	131	C	CTX-M-14	NAL FOF	This study

a Resistances against betalactam antibiotics are not included here. NAL = nalidixic acid, CIP = ciprofloxacin, SXT = trimethoprim-sulfamethoxazole, TOB = tobramycin, GEN = gentamicin, AMC = amoxicillin/clavulanic acid, FOF = fosfomycin.

### Pasteur Institute MLST Scheme

Eighteen of 23 isolates were characterized by gene amplification and sequencing of the eight housekeeping genes (*dinB, icdA, pabB, polB, putP, trpA, trpB* and *uidA*) by use of the protocol and primers specified at the Pasteur Institute website (http://www.pasteur.fr/recherche/genopole/PF8/mlst/EColi.html). The remaining 5 isolates had already been characterized [8].

### *fimH* Subtyping

A 489-nucleotide internal fragment of *fimH* (encoding the type 1 fimbrial adhesin) was amplified and sequenced to define clonotypes as described by Weissman et al. [20].

### *Xba*I PFGE Analysis

PFGE profiles were analyzed with the BioNumerics fingerprinting software (Applied Maths, ST-Martens-Latern, Belgium). Cluster analysis of the Dice similarity indices based on the unweighted-pair group method using average linkages (UPGMA) was done to generate a dendrogram describing the relationship among the PFGE profiles.

### Mouse Lethality Assay

A mouse sepsis model was used to assess the intrinsic extraintestinal virulence of the 23 ST131 human clinical isolates as described elsewhere [16]. Briefly, for each isolate, 10 outbred female RjOrl:Swiss mice (3–4 weeks old) received a subcutaneous injection into the nape of the neck of approximately 2×10^8^ CFU of log-phase bacteria. Previously, the isolates were isolated in LB agar (Invitrogen) (22 h) and then one colony was incubated with shaking (180 r.p.m.) at 37°C in 10 mL of Luria Broth (Invitrogen) (24 h). Cultures were centrifuged at 3,500 r.p.m for 15 min, washed twice with saline solution, and resuspended in saline solution again. The absorbance of suspensions were measured at 450 nm and adjusted to 1.4. These adjusted suspensions were used to inoculate the mice subcutaneously. After inoculation, the mice (which had free access to food and water) were observed for up to 1 week. Time of death and local presence of lesions (acute inflammation in the region of inoculation) were recorded for each mouse. Surviving mice were euthanatized on day 7 and their status were also recorded. In each lethality assay, two control isolates were included: K-12 MG1655 isolate, which does not kill mice by 7 days postchallenge, and CFT073 isolate which shows a lethality of ≥80% by 7 days postchallenge [11]. Control isolates were prepared in the same conditions as test isolates and injected in 5 mice each. With the purpose of comparing virulence potential of isolates, results of lethality were considered as: a) Number of mice dead within 24 h postinjection, being isolates classified as rapidly lethal if killed ≥80% of mice challenged within 24 h postinjection. b) Number of mice dead within 25–48 h postinjection. c) Total number of mice dead within 7 days of experiment (final lethality). Additionally, final lethality plus number of mice with local presence of lesions (acute inflammation in the region of inoculation) when euthanatized was also considered as virulence potential of isolates.

### Statistical Analysis and Factorial Analysis of Correspondence (FAC)

Comparisons of proportions were tested using Fisheŕs exact test. The criterion for statistical significance was set at a *P* value of <0.05.

A FAC was used to describe associations among the data [21]. FAC uses a covariance matrix based on chi2 distances. The computation determines a plane defined by 2 or 3 principal axes of the analysis; the first axis, F1, accounts for most of the variance, and the second or third axis, F2 or F3, which are orthogonal to F1, account for the largest part of the variance not accounted by F1. FAC was conducted with SPAD.N software (Cisia, Saint Mandé, France) from a two-way table. This table had 23 rows, corresponding to the 23 *E. coli* studied strains and 48 columns corresponding to 48 variables: the serotypes (O25b:H4 and O16:H5), the Pasteur Institute sequence types, the 9 virotypes and subtypes, the lethality in the mouse model of 10 mice in less than 24 hours, the lethality in the mouse model according to 3 categories (killer = 8 to 10 mice killed, intermediate killer = 3 to 7 mice killed, and non killer = 0 to 2 mice killed), the local presence of lesion (acute inflammation) in at least 5 mice, the high (≥18) or low (<17) virulence score, and the ExPEC-associated virulence genes detected in isolates. For each isolate, each variable was coded as present = 1, absent = 0.

## Results

### Virulence Genotypes and Plylogenetic Relationships

Table 2 shows the proposed virotype scheme based on the presence or absence of certain extraintestinal virulence genes, according to which the 23 ST131 human clinical isolates belonged to five main virotypes: the four described previously (A, B, C, D) [10] plus a new one described in the study of Dahbi et al. [8] and named now as virotype E. Additionally, virotype D was subdivided as D1, D2, D3, D4 and D5 due to the virulence-gene differences observed within it. Isolates belonging to serotype O16:H5 showed virotype C.

**Table 2:** Virulence-gene scheme for defining ST131 *E. coli* virotypes.

Virotype	Virulence genes^a^										
	*ibeA*	*iroN*	*sat*	*afa/draBC*	*afa* operon FM955459	*papG*II	*papG*III	*cnf1*	*hlyA*	*cdtB*	*neuC* K1
A	−	−	+/−b	+	+	−	−	−	−	−	−
B	−	+	+/−b	−	−	−	−	−	−	−	−
C	−	−	+	−	−	−	−	−	−	−	−
D1	+	+/−	−	−	−	−	−	−	−	+	−
D2	+	+/−	−	−	−	−	+	−	−	+	−
D3	+	+/−	+/−c	+/−c	+/−c	−	−	−	−	−	−
D4	+	+/−	−	−	−	−	−	−	−	−	+
D5	+	+/−	−	−	−	−	+	+	+	−	−
E	−	−	+	−	−	+	−	+	+	−	−

a Positive and negative results obtained by PCR for *ibeA* (invasion of brain endothelium), *iroN* (catecholate siderophore receptor), *sat* (secreted autotransporter toxin), *afa/draBC* (Afa/Dr adhesins), *afa* operon FM955459, *papG* II (allele II of *papG* gene), *papG* III (allele III of *papG* gene), *cnf1* (cytotoxic necrotizing factor type 1), *hlyA* (alpha-hemolysin), *cdtB* (cytolethal distending toxin) and *neuC*-K1 (K1 variant of group II capsule).

b Most isolates of virotype A and B are *sat*-positive.

c Isolates of virotype D3 carry genes *sat* and *afa*/*draBC*, or at least one of them. In addition, some *afa/draBC* strains are positive for *afa* operon FM955459.

**Figure 1:**
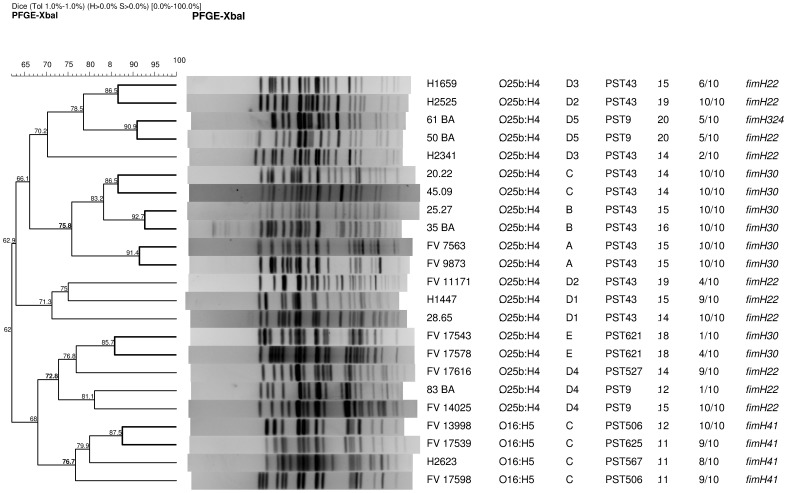
Pulsed-field gel electrophoresis of *Xba*I-digested DNA from 23 ST131 *E. coli* isolates. The dendrogram was obtained with the UPGMA algorithm based on the Dice similarity coefficient and applying 1% of tolerance in the band position. Isolate designation, O:H serotype, virotype, PST, virulence-gene score, final lethality (mice dead within 7 days/mice challenged), and *fimH* subtype are shown on the right.

According to the Pasteur Institute MLST scheme, the 23 ST131 isolates showed 7 different sequence types (identified as PST) (Table 3, Figure 1). O25b:H4-ST131 isolates of virotypes A, B and C showed PST43, while isolates of virotype D belonged to three different STs (PST9, PST43 and PST527), and isolates of virotype E belonged to PST621. O16:H5-ST131 isolates showed specific STs (PST506, PST567 and PST625).

**Table 3:** Allele profile of the 23 ST131 *E. coli* isolates by the Pasteur Institute MLST scheme.

PST	*dinB*	*icdA*	*pabB*	*polB*	*putP*	*trpA*	*trpB*	*uidA*	Serotype	Virotypes^a^
**43**	9	1	15	7	4	9	6	9	O25b:H4	A, B, C, D1, D2, D3
**9**	9	20	15	7	4	9	6	9	O25b:H4	D4, D5
**527**	9	20	15	7	4	9	6	129	O25b:H4	D4
**621**	9	234	15	7	4	9	6	9	O25b:H4	E
**506**	9	134	74	134	4	72	1	9	O16:H5	C
**567**	9	20	74	134	4	72	1	9	O16:H5	C
**625**	9	134	74	134	4	162	1	9	O16:H5	C

a Virotypes in which the indicated PST was detected.

Insertion of ISL*3*-like transposase gene in *fimB* was present in O25b:H4-ST131 isolates of virotypes A, B, C and E, but in none of virotype D (independently of the subtype). The *fimB* insertion was not detected in the four O16:H5-ST131 isolates either (Table 4).

**Table 4:** Associations of molecular types, virulence-gene profiles and virulence patterns in the murine sepsis model of the 23 ST131 *E. coli* isolates studied.

Isolate code	Serotype	Viro Type^a^	PST	Lethality^b^ ≤24 h	Lethality^c^ 25 h–48 h	Final lethality^d^	Final lethality+lesion^e^	Complete virulence-gene profile^f^	VG^g^ score	ExPEC status^h^
FV 9873	O25b:H4	A	43	10	0	**10**	10	*fimH30,* ISL*3-*like in *fimB,* F10 *papA, afa/draBC* i, *iha, sat, iucD, iutA, fyuA, chuA, kpsM II-*K2, *traT, malX, usp, ompT*	15	Yes
FV 7563	O25b:H4	A	43	10	0	**10**	10	*fimH30,* ISL*3*-like in *fimB,* F10 *papA, afa/draBC* i, *iha, sat, iucD, iutA, fyuA, chuA, kpsM II-*K2, *traT, malX, usp, ompT*	15	Yes
35 BA	O25b:H4	B	43	2	5	**10**	10	*fimH30,* ISL*3*-like in *fimB,* F10 *papA, iha, sat, iucD, iutA, iroN, fyuA, chuA, kpsM II-*K5, *iss, traT, malX, usp, ompT*	16	Yes
25.27	O25b:H4	B	43	10	0	**10**	10	*fimH30,* ISL*3-*like in *fimB,* F10 *papA, iha, sat, iucD, iutA, iroN, fyuA, chuA, kpsM II-*K5, *iss, malX, usp, ompT*	15	Yes
20.22	O25b:H4	C	43	10	0	**10**	10	*fimH30,* ISL*3-*like in *fimB,* F10 *papA, iha, sat, iucD, iutA, fyuA, chuA, kpsM II-*K5, *traT, malX, usp, ompT*	14	Yes
45.09	O25b:H4	C	43	4	4	**10**	10	*fimH30,* ISL*3-*like in *fimB,* F10 *papA, iha, sat, iucD, iutA, fyuA, chuA, kpsM II-*K5, *traT, malX, usp, ompT*	14	Yes
28.65	O25b:H4	D1	43	2	8	**10**	10	*fimH22, cdtB, iucD, iutA, fyuA, chuA, kpsM II-*K5, *iss, traT, ibeA, malX, usp, ompT*	13	Yes
H1447	O25b:H4	D1	43	3	6	**9**	9	*fimH22, cdtB, iucD, iutA, fyuA, chuA, kpsM II-*K5, *iss, traT, ibeA, malX, usp, ompT*	13	Yes
FV 11171	O25b:H4	D2	43	0	0	**4**	9	*fimH22, papAH, papC, papEF, papG* III, *cdtB, iucD, iutA, iroN, fyuA, chuA, kpsM II-*K5, *cvaC, iss, traT, ibeA,* *malX, usp, ompT*	19	Yes
H2525	O25b:H4	D2	43	2	8	**10**	10	*fimH22, papAH, papC, papEF, papG* III, *cdtB, iucD, iutA, iroN, fyuA, chuA, kpsM II-*K5, *cvaC, iss, traT, ibeA,* *malX, usp, ompT*	19	Yes
H1659	O25b:H4	D3	43	0	3	**6**	7	*fimH22,* F10 *papA, afa/draBC* i, *iha, sat, iucD, iutA, fyuA, chuA, kpsM II-*K5, *traT, ibeA, malX, usp,ompT*	15	Yes
H2341	O25b:H4	D3	43	0	1	**2**	4	*fimH22,* F10 *papA, iha, sat, iucD, iutA, fyuA, chuA, kpsM II-*K5, *traT, ibeA, malX, usp, ompT*	14	Yes
FV 14025	O25b:H4	D4	9	3	7	**10**	10	*fimH22, iucD, iutA, iroN, fyuA, chuA, kpsM II-*K1, *cvaC, iss, traT, ibeA, malX, usp, tsh, ompT*	15	Yes
83 BA	O25b:H4	D4	9	0	0	**1**	8	*fimH22, iha, iroN, fyuA, chuA, kpsM II-*K1, *iss, traT, ibeA, malX, usp, ompT*	12	No
FV 17616	O25b:H4	D4	527	5	4	**9**	9	*fimH22, iucD, iutA, iroN, fyuA, chuA, kpsM II-*K1, *cvaC, iss, traT, ibeA, malX, usp, ompT*	14	Yes
61 BA	O25b:H4	D5	9	4	1	**5**	5	*fimH324, papAH, papC, papEF, papG* III, *cnf1, hlyA, iucD, iutA, iroN, fyuA, chuA, kpsM II-*K5, *cvaC, iss, traT,* *ibeA, malX, usp, ompT*	20	Yes
50 BA	O25b:H4	D5	9	0	0	**5**	5	*fimH22, papAH, papC, papEF, papG* III, *cnf1, hlyA, iucD, iutA, iroN, fyuA, chuA, kpsM II-*K5, *cvaC, iss, traT,* *ibeA, malX, usp, ompT*	20	Yes
FV17578	O25b:H4	E	621	0	1	**4**	10	*fimH30,* ISL*3-*like in *fimB,* F10 *papA, papEF, papG* II, *iha, sat, cnf1, hlyA, iucD, iutA, fyuA, chuA, kpsM II-*K5,*traT, malX, usp, ompT*	18	Yes
FV 17543	O25b:H4	E	621	0	1	**1**	10	*fimH30,* ISL*3-*like in *fimB,* F10 *papA, papEF, papG* II, *iha, sat, cnf1, hlyA, iucD, iutA, fyuA, chuA, kpsM II-*K5,*traT, malX, usp, ompT*	18	Yes
FV 17539	O16:H5	C	625	0	5	**9**	10	*fimH41,* F10 *papA, sat, iucD, iutA, fyuA, chuA, traT, malX, usp, ompT*	11	No
FV 17598	O16:H5	C	506	0	5	**9**	10	*fimH41,* F10 *papA, sat, iucD, iutA, fyuA, chuA, traT, malX, usp, ompT*	11	No
H2623	O16:H5	C	567	0	8	**8**	10	*fimH41,* F10 *papA, sat, iucD, iutA, fyuA, chuA, traT, malX, usp, ompT*	11	No
FV 13998	O16:H5	C	506	3	7	**10**	10	*fimH41,* F10 *papA, sat, iucD, iutA, fyuA, chuA, kpsM II-*K2, *traT, malX, usp, ompT*	12	Yes

a Virotypes (including new assigned E) and subtypes of virotype D.

b Number of mice dead within 24 h postinjection. Isolates were classified as rapidly lethal if ≥80% of mice challenged with them died within 24 h postinjection.

c Number of mice dead within 25–48 h postinjection.

d Total number of mice dead within 7 days of experiment.

e Total number of mice dead within 7 days of experiment (final lethality) plus number of mice with local presence of lesion (acute inflammation in the region of bacterial inoculation) when euthanatized.

f Virulence-gene profile obtained by PCR for detection of 40 ExPEC-associated virulence genes.

g Virulence-gene (VG) score calculated as the sum of all virulence genes detected in an isolate.

h ExPEC status defined as presence ≥2 of *papEF*, *sfa/focDE*, *afa/draBC*, *iutA* and *kpsM II*.

i Isolates FV 9873, FV 7563 and H1659 exhibited *afa* operon FM955459.

Four different *fimH* alleles were identified. O25b:H4-ST131 isolates of virotypes A, B, C and E carried *fimH30*. All O25b:H4-ST131 isolates of virotype D contained *fimH22* but one, which showed *fimH324* (only one nucleotide difference with *fimH22*). The four O16:H5-ST131 isolates of virotype C carried *fimH41* (Table 4).

Overall, the 19 ST131 isolates of serotype O25b:H4 exhibited higher virulence scores (mean, 15.7; range, 12 to 20) than ST131 isolates of serotype O16:H5 (mean, 11.2; range, 11 to 12). Besides, 3 of 4 O16:H5-ST131 isolates and 1 O25b:H4-ST131 isolate of virotype D4 did not show ExPEC status (Table 4).

The PFGE dendrogram obtained from the 23 *Xba*I-digested ST131 isolates showed high heterogeneity (<65% identity). Six clusters of similarity >85% grouped O25b-ST131 (2 isolates each) of virotypes A, B, C, D and E. Isolates of subtypes D1 to D5 disaggregated in different branches of the dendrogram. The 4 O16:H5-ST131 isolates remained grouped in the dendrogram with 76.7% identity (Figure 1).

### Mouse Sepsis Model

In the mouse sepsis model, within the 7 dayś experiment, each 4 or all 5 mice challenged with positive-control isolate CFT703 died, compared with none of 5 mice challenged with negative control isolate MG1655, which remained completely healthy. The lethality results observed for CFT703 and MG1655 isolates were similar within the 3 test lots in which the 23 ST131 isolates were assayed. Thus, fourteen of 15 (93%) mice challenged with CFT703 died (9 of 15, 60% of mice dead within 24****h; and 5 of 15, 33% within 25–48****h of inoculation) *versus* none of 15 (0%) mice challenged with MG1655.

Overall, 14 out of the 23 ST131 isolates tested (61%) killed 90 to 100% of mice challenged, and 18 of 23 isolates (78%) at least 50% of mice. Different virulence patterns were observed in the number of total deaths (final lethality), the rapidity (lethality ≤24****h, 25–48****h, >48****h) and the inflammation-causing ability of the isolates. Furthermore, variability was observed even within certain virotypes (Table 4). Thus, the highest lethality (final lethality ≥80% of mice challenged killed) was shown by all 8 O25b:H4 isolates of virotypes A, B, C and D1, and 4 O16:H5 isolates of virotype C. By contrast, isolates within virotypes D2 and D4 had very different outcomes (4 mice *versus* 10, virotype D2; 10 and 9 mice *versus* 1, virotype D4); and isolates of virotype E showed the lowest final lethality.

Rapid lethality (≥80% of mice dead within 24****h of inoculation) was shown by both O25b:H4 isolates of virotype A, and 1 O25b:H4 isolate of virotypes B and C, respectively.

Certain ST131 isolates within virotypes D, E (O25b:H4) and C (O16:H5) induced an acute inflammatory response in mice, in the neck region. This finding was recorded as number of mice with presence of lesions (Table 4). And considering as potentially virulent isolates those exhibiting lethality and/or with inflammation-causing ability, 19 of the 23 ST131 isolates (83%) showed a value for this parameter (final lethality+lesion) of ≥80% of mice challenged. Only O25b:H4 isolates of virotypes D3 and D5 showed a value of <80% (Table 4).

Although no significant difference in lethality was found between highly virulent ST131 O25b:H4 isolates of virotypes A, B, C and O16:H5 isolates of virotype C, the latter killed mice less rapidly. Significant differences were found, however, between isolates of virotypes A, B, C (final lethality ≥80% of mice challenged) and isolates of virotypes D, E (*P*<0.01).

### Factorial Analysis of Correspondence

**Figure 2:**
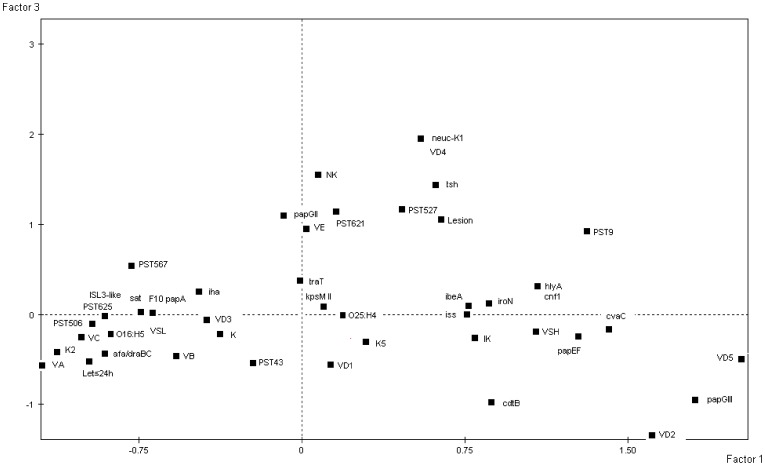
Projections of the serotypes (O25:H4 and O16:H5), the 7 PSTs (PST9, PST43, PST506, PST525, PST567, PST621 and PST625), the 9 virotypes (VA, VB, VC, VD1, VD2, VD3, VD4, VD5 and VE), the mouse lethality occurring in less than 24 hours (Let ≤24 h), the lethality in the mouse model as killer (K), intermediate killer (IK) and non-killer (NK), the local presence of acute inflammation (lesion), the high (VSH) or low (VSL) virulence score and 25 virulence genes (listed as in Table 4) characterising the 23 *E. coli* strains on the plane F1/F3 computed from the factorial analysis of correspondence. Only one square is indicated when several variables are projected at the same position.

To have an overview of the associations between all the data, a FAC was conducted. This approach is particularly well suited to the analysis of small size of individuals with numerous variables. It captures global relationships between variables, the projection of these variables on the plane being the result of the multiple associations between them. The projection of the variables on the plane F1/F3, which accounted for 34.52% of the total variance, distinguished the lethality in less than 24****h, the killer phenotype, the virotypes A, C, B and D3, the PSTs 506, 625, 567 and 43, the serotype O16:H5, the low score of virulence genes, and the virulence genes K2, *afra/draBC*, ISL*3*-like in *fimB, sat*, F10 *papA*, *iha* on the negative values of the first factor F1 from intermediate killer phenotype, the virotypes D1, 2 and 5, the PST9, the serotype O25:H4, the high score of virulence genes, and a large number of virulence genes which were projected on the positive values of this factor. The third factor F3 distinguished the non-killer phenotype and the local presence of lesion (acute inflammation) on the positive values with the virotypes E and D4, the PSTs 621 and 527, and the virulence genes *papG*II, *neuC* and *tsh* (Figure 2). Collectively, this analysis confirms that there is (i) a high heterogeneity of the virulence-gene content with some associations of PST/serotype with specific patterns of presence/absence of virulence genes corresponding to virotypes and (ii) a strong association between specific virotypes and virulence in mice, *i.e.* virotypes A, B and C being highly virulent whereas virotype E is non-lethal but associated to an acute inflammatory response. This association is not due to the total number of virulence genes but seems the result of specific combinations of them.

## Discussion

In the assessment of 23 ST131 *E. coli* clinical isolates belonging to 2 serotypes (O25b:H4 and O16:H5) and to nine virotypes and subtypes (A, B, C, D1, D2, D3, D4, D5 and E) for extraintestinal virulence in a murine sepsis model, we have found a wide range of mouse virulence patterns with association of certain virotypes.

In a preliminary study of virulence in murine sepsis model, Clermont et al. [13] studied 4 ST131 isolates which all yielded 100% lethality, suggesting that rapid and extensive emergence of CTX-M-15-producing ST131 clonal group might be due to its high level of antibiotic resistance and its high virulence-gene scores. However, a subsequent study including ST131 (27 isolates) and non-ST131 (34 isolates) clinical *E. coli* concluded that ST131 isolates are neither uniformly virulent nor, as a group, discernibly more virulent than other extraintestinal *E. coli* isolates. In that study, even 8 out of the 27 ST131 isolates (30%) did not kill any mouse within 7 days (0% of final lethality) [14]. In accordance with Johnson et al. [14], we have found broad virulence diversity among ST131 clinical isolates in the mouse sepsis model. But differently to their outcomes, all our 23 tested ST131 isolates exhibited some lethality for mice (none isolate showed 0% of lethality). Furthermore, 18 of our 23 ST131 isolates (78%) killed at least 50% of mice challenged *versus* 12 of 27 (44%) in the study of Johnson et al. [14] (*P* = 0.01). These discrepancies observed in the mouse sepsis model between the two studies could be due to the differences within both ST131 collections. Thus, only 5 of the 27 ST131 isolates (19%) of Johnson et al. [14] were ESBL-producers *versus* 18 of 23 (78%) of the present study (*P*<0.01).

Interestingly, we have observed virulence patterns associated to certain virotypes. ST131 isolates within virotypes A, B and C showed uniform results with high scores of final lethality (≥80%), being both O25b:H4 isolates of virotype A those with more rapid lethality (100% of mice died within 24****h) in repeated assays (data not shown). In fact, isolates of virotype A killed even more rapidly than positive control CFT073 (60% of mice died within 24****h). On the contrary, O25b:H4 isolates of virotype D exhibited variable virulence patterns even within the subtypes D2, D3 and D4, and O25b:H4 isolates of virotype E showed a pattern of slow and low lethality with inflammation-causing ability (presence of lesion). This *in vivo* virulence diversity within ST131 clonal group would reflect the genetic variability evidenced by molecular typing of the housekeeping genes that defines PSTs, the *fimH* subtyping that defines clonotypes, and the PFGE profiles obtained from *Xba*I-digested DNA. Thus, we found that O25b:H4 isolates of virotypes A, B and C showed the same ST (PST43), same *fimH30* subtype and their PFGE patterns grouped together in dendrogram (75.8% similarity). O25b:H4 isolates of virotype D belonged to three different STs (PST9, PST43 and PST527), two *fimH* subtypes (22 and 324) and distributed in distant branches of the dendrogram. O25b:H4 isolates of virotype E belonged to PST621, showed *fimH30* and grouped with O25b:H4 D4 isolates (72.8% similarity). And finally, O16:H5 isolates of virotype C showed specific STs (PST506, PST567 and PST625), specific *fimH41* and remained grouped with 76.7% identity.

This is the first study to assess virulence of ST131 isolates of serotype O16:H5, which in spite of their low virulence-gene score, did not show significant differences in final lethality with highly virulent O25b:H4 isolates of virotypes A, B and C, but killed less rapidly. High virulence of O16:H5 ST131 isolates in the mouse model would correlate with clinical severity reported by Kudinha et al. [22], [23] who found a significant association of O16 ST131 with pyelonephritis in men and reproductive-age women. To our knowledge, this is also the first report of clonotype H41 in relation with serotype O16:H5. Clonotyping (combination of *fumC* and *fimH*_TR_ sequences) is an important tool for the discrimination of sublineages within extraintestinal pathogenic *E. coli* clones. Furthermore, a recent study reports that specific clonotypes correlate with antimicrobial susceptibility profiles and clinical outcomes [24]. In our study, we have identified 4 *fimH* subtypes correlated with serotype and virotype: clonotypes H22, H324 (O25b:H4 virotype D), H30 (O25b:H4 virotypes A, B, C and E) and H41 (O16:H5 virotype C). H30 clonotype, which comprises almost all current fluoroquinolone-resistant ST131 isolates, has expanded more extensively than other ST131 variants [24],[25]. This clonotype has been significantly correlated with recurrent and persistent urinary tract infection and clinical sepsis [24], which would be in accordance with our finding of high lethality in the mouse model exhibited by O25b:H4 isolates of virotypes A, B and C (*fimH30*). Surprisingly, O25b:H4 isolates of virotype E belonging to clonotype H30 and with high virulence-gene score showed low lethality in the mouse model, and differently to virotypes A, B and C, O25b:H4 isolates of virotype E show PST621. Therefore, O25b:H4 of virotype E would represent a subclone within clonotype H30.

Blanco et al. [10] also found that certain epidemiological and clinical features corresponded with the virotype, which might involve the action of different mechanisms of pathogenesis explaining at least partially different patterns of virulence exhibited by ST131 virotypes.

Lavigne et al. [15] assessed the virulence of 3 ST131 isolates compared with 5 non-ST131 in two alternative animal models (nematode *Caenorhabditis elegans* and zebrafish embryos). According to our virotype scheme, the 3 O25b:H4 ST131 included in their study belonged to virotypes A, B (both CTX-M-15 isolated from cystitis) and D (an isolate obtained from stools of a healthy subject), respectively. Results obtained in those animal models were very different to ours in mouse. Particularly, ST131-B2 isolates were less virulent than non-ST131 B2 isolates in the nematode model. In zebrafish, the 2 CTX-M-15-producing ST131 UPEC isolates were also less virulent than the non-ST131 B2 strains, but O25b:H4 strain of virotype D (S250) showed higher virulence than the two other O25b:H4 ST131 strains (virotypes A and B). Authors suggested that their findings of low virulence for ST131 isolates could be explained by the absence of several classical virulence factors such as HlyA and Cnf1. Results reported by Lavigne et al. [15] are hardly comparable with ours because of the different model used. In the mouse model, O25b:H4 isolates of virotypes A and B showed high virulence. Furthermore, we found that virotype D was significantly less virulent that virotypes A, B and C. In our study, we have not found association of *hlyA* and *cnf1* presence and virulence in mouse model. In fact, O25b:H4 isolates of virotypes D5 and E positive for those genes and exhibiting the highest virulence-gene scores (20 and 18, respectively) showed moderate to low lethality (≤50% of mice challenged).

The non-correlation of the virulence in mice with the virulence score within the ST131 isolates is different to what has been observed at the species level [11], [16]. This indicates that in a specific genetic background, *i.e.* the ST131, virulence is more linked to specific combinations of virulence genes than to the total number of them (Figure 2), which could correspond to epistasis between the different portions of the genome. And as already pointed out by other authors [11], [26], there is an urgent need to discover the presumed unidentified accessory traits responsible of within-clonal group variation. It is also important to bear in mind that limitations of the present study include the mouse model used. Subcutaneous infection does not reproduce UTI or bacteremia developed in humans by the clinical ST131 isolates tested. So, sepsis model may not reflect essential virulence genes for adhesion, invasion and/or dissemination.

### Conclusion

This study has evidenced the high genetic variability within the ST131 clonal group, also reflected in the lethality results of a mouse sepsis model. We have shown that Pasteur Institute MLST scheme, as well as clonotyping, are important molecular tools for the discrimination of sublineages within ST131 clonal group which exhibited *in vivo* virulence patterns associated to specific virotypes.
